# On Collodion as a Means of Arresting Hæmorrhage from Leech-bites

**Published:** 1849-02

**Authors:** 


***On Collodion as a means of arresting Hæmorrhage from Leech-bites.***

To the Editor of the London Lancet.

Sir,—May I beg a place in your excellent journal for the accompanying letter, addressed to me by Mr. Tucker, as a report on the efficacy of collodion in a case of hæmorrhage from leech-bites. It will be seen, on a perusal of the letter, that Mr. Tucker felt some difficulty in managing the compress, in consequence of its adhesion to his finger as well as to the wound, and as this difficulty may perchance be experienced by others, I beg to suggest that the compress should be covered by a little disc of thin paper as soon as applied, and that the latter should be pressed with the finger, or with any instrument calculated to make direct pressure, such as the head of a pencil or pencil-case. In this way, the possibility of adhesion between the application and the object making the pressure would be effectually obviated, and every purpose gained. I need hardly observe, that the adaptation of the collodion made by Mr. Tucker is not among the least valuable of its purposes. I have the honour to be, Sir, your obedient servant, ERASMUS WILSON.

*Berners-street, December 2d,* 1848.

MY DEAR SIR,—When reading your article on the uses of collodion, in THE LANCET for November 18th, it struck me that the solution might be rendered available in hæmorrhages from leech-bites, (some of which, most medical men know, are difficult of arrest,) and I resolved to give it a trial. An opportunity occurred on the 30th of November. I called to see a young female, to whose temples I had ordered four leeches on the previous night, and found blood escaping then very freely, a period of twelve hours from their application; the countenance was blanched, and my patient expressed herself as being faint. On removing from the forehead the napkin, which was completely saturated, blood trickled copiously down the cheeks from one orifice, apparently from the wounding of a small branch of the temporal artery.

My patient residing close to your residence, I thought I would avail myself of your opinion as to the efficacy of collodion in this case, and was pleased with finding that you did not consider this simple affair unworthy your notice, and with your readiness to furnish me with the remedy. I will now state the result.

As you advised, I took with my forceps some of the flocculent portion of the lint dipped in the collodion, applied it to the orifice, and pressed it with my finger. The blood continued to flow, as I had not placed the finger exactly on the orifice. The second trial was more successful: I allowed the finger to remain till I supposed the lint had adhered: on attempting to remove my finger cautiously, I found it a fixture, but with the point of the forceps I separated the fibres of the lint from it; blood still continued to flow, but in a less degree, from the lowest point. Without removing the first, I applied a second piece of lint, dipped in the solution as before, which soon became adherent, and stopped the bleeding; in another moment I found blood oozing from above; another similar application arrested it. I then, as you advised, with a camel-hair pencil, applied the collodion freely around the edges, and over the whole applications. I saw my patient in the evening, no blood had escaped, nor did she complain of any pain or undue pressure.

I prefer this mode of arresting hæmorrhages from leech-bites to any I have ever tried; and as I have witnessed a case of death in a child from the bite of a single leech, and have heard of others, I attach much importance to the use of collodion in such hæmorrhages. I am confident my patient must have lost sixteen ounces of blood.—Truly yours, J. H. Tucker.

To Erasmus Wilson, Esq.

